# Quality of anticholinergic burden scales and their impact on clinical outcomes: a systematic review

**DOI:** 10.1007/s00228-020-02994-x

**Published:** 2020-10-03

**Authors:** Angela Lisibach, Valérie Benelli, Marco Giacomo Ceppi, Karin Waldner-Knogler, Chantal Csajka, Monika Lutters

**Affiliations:** 1 Clinical Pharmacy, Department Medical Services, Cantonal Hospital of Baden, Baden, Switzerland; 2 Department of Neurorehabilitation, RehaClinic, Bad Zurzach, Switzerland; 3 Basel Pharmacoepidemiology Unit, Division of Clinical Pharmacy and Epidemiology, Department of Pharmaceutical Sciences, University of Basel, Basel, Switzerland; 4 Qualitätszentrum für Medikamentensicherheit, Mediq, Brugg, Switzerland; 5 Center for Research and Innovation in Clinical Pharmaceutical Sciences, Institute of Pharmaceutical Sciences of Western Switzerland, University Hospital and University of Lausanne, Lausanne, Switzerland; 6 School of Pharmaceutical Sciences, Institute of Pharmaceutical Sciences of Western Switzerland, University of Lausanne, University of Geneva, Geneva, Switzerland; 7 Swiss Federal Institute of Technology, Zurich, Switzerland

**Keywords:** Cumulative anticholinergic burden, Clinical outcomes, Older people, Quality assessment, Validation

## Abstract

**Purpose:**

Older people are at risk of anticholinergic side effects due to changes affecting drug elimination and higher sensitivity to drug’s side effects. Anticholinergic burden scales (ABS) were developed to quantify the anticholinergic drug burden (ADB). We aim to identify all published ABS, to compare them systematically and to evaluate their associations with clinical outcomes.

**Methods:**

We conducted a literature search in MEDLINE and EMBASE to identify all published ABS and a Web of Science citation (WoS) analysis to track validation studies implying clinical outcomes. Quality of the ABS was assessed using an adapted AGREE II tool. For the validation studies, we used the Newcastle-Ottawa Scale and the Cochrane tool Rob2.0. The validation studies were categorized into six evidence levels based on the propositions of the Oxford Center for Evidence-Based Medicine with respect to their quality. At least two researchers independently performed screening and quality assessments.

**Results:**

Out of 1297 records, we identified 19 ABS and 104 validations studies. Despite differences in quality, all ABS were recommended for use. The anticholinergic cognitive burden (ACB) scale and the German anticholinergic burden scale (GABS) achieved the highest percentage in quality. Most ABS are validated, yet validation studies for newer scales are lacking. Only two studies compared eight ABS simultaneously. The four most investigated clinical outcomes delirium, cognition, mortality and falls showed contradicting results.

**Conclusion:**

There is need for good quality validation studies comparing multiple scales to define the best scale and to conduct a meta-analysis for the assessment of their clinical impact.

**Electronic supplementary material:**

The online version of this article (10.1007/s00228-020-02994-x) contains supplementary material, which is available to authorized users.

## Introduction

Epidemiologic studies have shown that 50% of the older population uses at least one drug with anticholinergic (ACH) properties. This is due to their use for multiple indications such as urinary incontinence or sleep disorders [1]. Furthermore, prescription increases with hospitalization [1–3]. Patients above the age of 65 years are at higher risk of experiencing ACH side effects due to physiological changes such as a decline in renal and liver function affecting drug elimination, changes in body mass distribution or increased blood-brain barrier (BBB) permeability [4–6]. ACH side effects are separated into peripheral (e.g. mouth dryness, blurred vision) and central (e.g. dizziness, mental confusion) side effects depending on the drug’s ability to pass the BBB. It has been shown that the increase in ACH side effects could result in negative clinical outcomes [3].

In 2001, Tune et al. defined the “anticholinergic burden” as a cumulative effect of taking one or more drugs susceptible to inducing ACH adverse effects [7]. It is important that clinicians have a valid method of measuring the ACH burden at their disposal to reduce such negative effects.

Presently, there is no gold standard for assessing the ACH burden in a patient. The two current major methods are the serum radioreceptor anticholinergic activity assay (SAA) and expert-based lists of medications with ACH properties, the so-called anticholinergic burden scales (ABS) or equations. The ABS generally assign a number from 0 (=no) to 3 (=high) to each substance according to its ACH properties. The first step in calculating the ACH burden of patients is done by identifying all prescribed ACH drugs followed by adding up the scores of each substance (cumulative ACH burden). The resulting score helps identifying patients at high risk of adverse events and provide guidance on interventions. Rudd et al. stated that these expert-based lists are the sole clinically useful tool to measure central ACH burden [8]. Apart from the expert-based lists are also equation-based approaches calculating the drug burden of a patient. Therefore, the drug burden is calculated using an equation, which includes daily-prescribed dosage and minimum recommended daily dosage and neglects ACH properties [9].

So far, we have identified five reviews describing ABS and their validation studies [6, 9–12]. However, these were descriptive reviews lacking systematic quality assessments for both the ABS and their validation studies. In this review, we aim to identify all published ABS and their validation studies, assess the quality of the ABS and their validation studies based on a systematic approach using adapted tools and evaluate their associations with clinical outcomes.

## Methods

### Search strategy and selection criteria

A systematic review was undertaken in accordance with the PRISMA statement [13]. We conducted a literature search in MEDLINE and EMBASE in March 2019 without date limitation but language restriction to German, French and English to identify all published ABS. The search was updated prior to the submission of this article to identify any new publication. The exact search queries for both databases are depicted in Appendix 1.

**Fig. 1:**
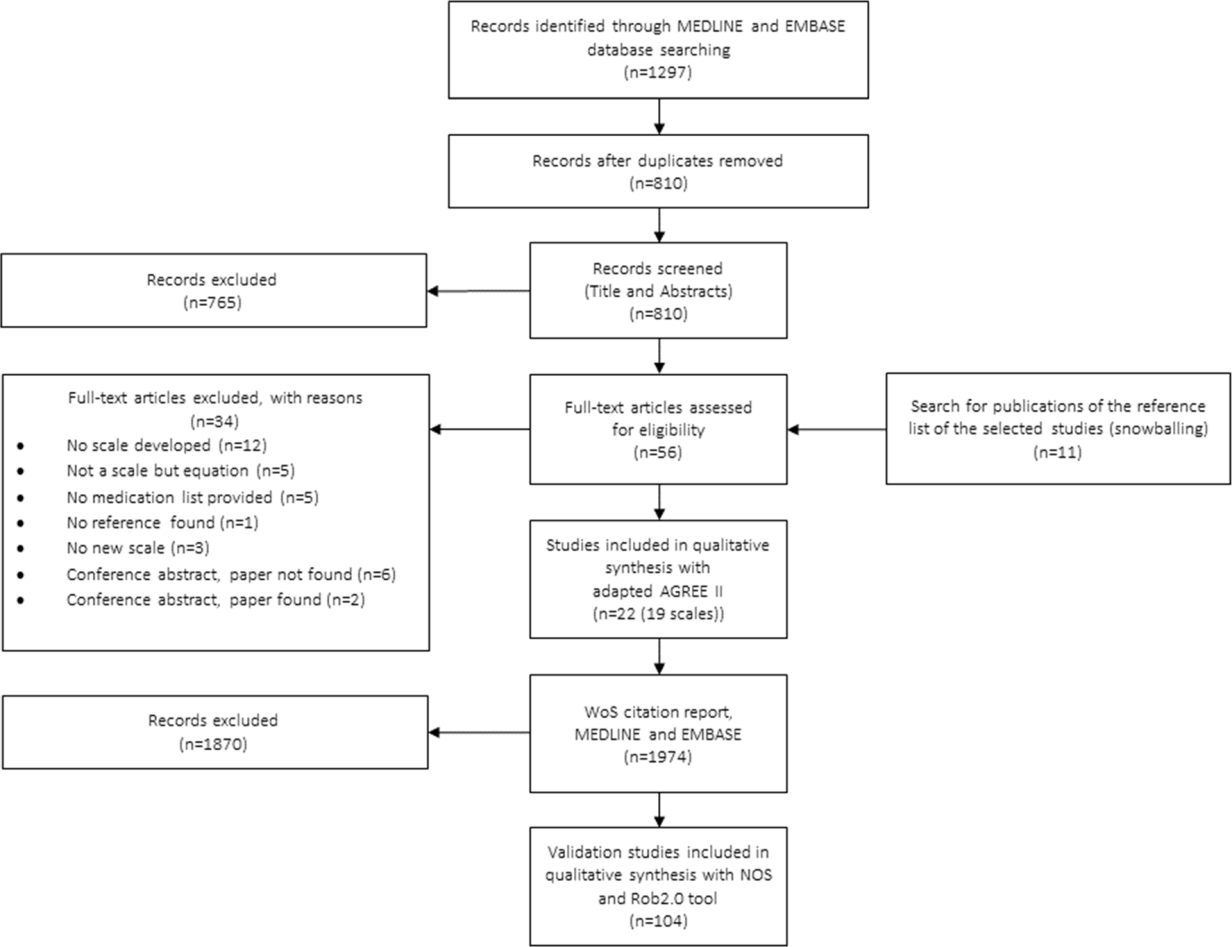
PRISMA flowchart. The identification of all published ABS and their validation studies (a detailed separate flowchart for the validation studies is depicted in Appendix 2)

Following the first literature search, a citation analysis was performed using Web of Science (WoS) to track validation studies for all identified ABS and relating them to clinical outcomes. Both searches were supplemented by manual searching of reference lists of the selected studies (snowballing). All found articles were imported to a citation manager (Endnote) and duplicates were removed. A flowchart of the search strategy is depicted in Fig. 1; a separate and detailed flowchart for the identification of all validation studies can be found in Appendix 2.

Inclusion criteria for the ABS were (1) existence of a grading score for each medication, (2) availability of a medication list and (3) development of the ABS for adults’ ≥ 18 years. We excluded ABS that were based on an equation calculating the ACH burden score for each substance. The corresponding authors were asked to provide an updated version of the included scales if no reference thereof could be found in the literature.

Inclusion criteria for the validation studies were (1) use of one of the identified ABS and calculation of the cumulative ACH burden, (2) evaluation of at least one clinical outcome, (3) inclusion of adults ≥ 18 years and (4) study designs: randomized controlled trial (RCT), cohort and case-control or cross-sectional studies. We excluded all validation studies that used the ABS to differentiate between users and non-users of ACH medication and study designs such as case reports, letters, pilot studies, case series, editorials and conference abstracts.

Two independent researchers (AL, VB) performed article screening and selection. Disagreements among the researchers were discussed with a third researcher (ML) until consensus was reached.

### Data extraction

Two researchers (AL, VB) independently extracted data onto a standardized spreadsheet. For the ABS, we extracted the following data: abbreviation, name of ABS, country/year, author, update, number of drugs scored, scoring levels, expert committee, description of scoring process or of resolving discordance among experts or previous lists and source of evidence (clinical expert opinions, dosage consideration, SAA/muscarinic receptor affinity, ACH adverse drug events, drug interaction, administration route, BBB permeability and based on prior published ABS). For the validation studies, we extracted the following data: author, title, year, used ABS, number of compared ABS, study design, study population, clinical outcome(s) and association studied.

### Quality assessment of scales

In a next step, we used the AGREE II tool to compare the ABS by systematically assessing their quality [14]. As the tool was originally developed and used for the quality assessment of clinical guidelines, we treated the ABS as individual guidelines. Prior to use, four researchers (AL, VB, KWK, ML) analyzed the tool independently and made suggestions for adaptions of the tool items. These were discussed and a consensus was reached. The adapted AGREE II tool can be found in Appendix 3. From 23 items, it was shortened to 18 items in 6 domains (deleted items from the Original AGREE tool II were number 5, 11, 16, 18, 20, 21). Furthermore, we added the item “Suggestions for further research” and changed “External review” into “Validation of scale”. For the item “Validation of scale”, we provided the researchers with a spreadsheet of all validation studies with their quality and evidence level as described below. Three researchers (AL, MGC, ML) independently assessed the quality of each ABS using the adapted AGREE II tool and scored each item from 1 (lowest) to 7 (highest). We also asked the researchers to provide an overall assessment score of the ABS and to state if they would recommend it for use. The analysis was performed according to the AGREE II tool manual with the exception that item 7 “Evidence selection criteria” and 11 “Validation of scale” were counted twice as they were considered more important. Absolute agreement of the three researchers was assessed by calculating the intraclass correlation coefficient (ICC) with a two-way model.

### Risk of bias of validation studies

In a next step, two researchers (AL, ML) independently appraised the quality of all validation studies using two published tools depending on the study design. For cohort, case-control and cross-sectional studies, we used the Newcastle-Ottawa Scale (NOS) [15] and for the RCT Risk of bias 2.0 (Rob2.0) from the Cochrane Collaboration [16]. We adapted the NOS for case-control studies by changing the “Non-response Rate” to “Missing Data”. For cross-sectional studies, we used the NOS for cohort studies by changing in *Selection “*3) Ascertainment of exposure” into “3) Measurement of method of exposure” and in *Outcome* “3) Adequacy of follow-up of cohorts” into “3) Missing data for cross-sectional”. Additionally for cross-sectional studies, we answered *Selection “*4) Demonstration that outcome of interest was not present at start of study or baseline measurement” and *Outcome* “2) Was follow-up long enough for outcomes to occur” always with a “no”. The forms can be found in the Appendix 4. The scores from the NOS and RoB2.0 were transformed into Agency for Healthcare Research and Quality standards (AHRQ) of “good”, “fair” or “poor” quality according to the conversion rules (Appendix 5). Considering the quality standards, we categorized each study into one of the evidence levels: 1 RCT (good and fair quality), 2a RCT (poor quality) and prospective cohort studies (good and fair quality), 2b retrospective cohort studies (good and fair quality), 3 case-control studies (good and fair quality), 4 cohort and case-control studies (poor quality) and 5 cross-sectional studies (good, fair and poor quality). The levels were based on the propositions of the Oxford Center for Evidence-Based Medicine [17]. Two researchers (AL, ML) independently assessed the quality and assigned evidence levels. Disagreements in assessment were resolved by discussion.

As the included studies were very heterogeneous, we refrained from conducting meta-analysis for clinical outcomes.

### Software use

Graphical demonstrations and calculations were performed in R Studio [18–22].

## Results

### Identification of published ABS and their validation studies

Out of 1297 records identified in the database and additional 11 records through snowballing, 24 studies describing 22 different ABS [23–46] were selected (Fig. 1). We excluded two studies by Aizenberg et al. [45] and by Whalley et al. [46], as no medication list was provided after contacting the authors, resulting in a total of 22 records describing 19 different ABS[23–44]. The updated search revealed no new scale. Our search query also identified the often used DBI (Drug Burden Index) and ACH-DBI [47, 48], the Drug Delirium Scale (DDS) [49], the scale by Cao [50] and the most recently developed MARANTE scale [51]. However, these five ABS were excluded as they were based on equations. The Summated Anticholinergic Medications Scale (SAMS) identified in the paper by Naples et al. [52] was excluded since no proper reference was found.

The 19 unique ABS (Table 1) arise from 11 different countries (USA, Thailand, Brazil, Germany, Korea, Canada, Norway, Ecuador, France, UK and Italy). Four scales (DS [32], GABS [38], KABS [37], BAADS [40]) have been elaborated by summarizing scores of previous published scales through an algorithm to develop a new score. For the GABS and the KABS, an expert committee scored some new drugs, while the other two did not state any expert committee nor scoring of new drugs. Most of the other ABS were developed by a literature research identifying ACH properties for each substance complemented by clinical expertise. These properties were mainly muscarinic receptor affinity, BBB permeability, drug interactions, ACH adverse events and serum radioreceptor anticholinergic activity assay (SAA). The Chew’s scale and the ATS were developed differently. While the Chew’s scale is based on an in vitro SAA measurement, the ATS is the only scale based on computational receptor binding affinity. Most ABS used a four-level grading system from 0 to 3 except for the DS [32] (high and low), the AAS [33] (5 levels), the Chew scale [31] (5 levels), the ATS [44] (continuous values), the SCDL [36] (3 levels) and the CI, PI (relative continuous values) [39]. Overall 787 different substances have been scored.

**Table 1:** Descriptive overview of all identified ABS

Abbreviation	Name of scale	Country / Year	Author	Update	Number of drugs scored	Scoring levels	Expert committee	Description of scoring process or of resolving discordance among experts or previous lists	Clinical expert opinions	Dosage consideration	SAA / Muscarinic Receptor Affinity	ACH adverse drug events	Drug interaction	Administration route	BBB permeability	Based on prior published scales
AAS	Anticholinergic Activity Scale	Norway /2010	Ehrt et al. [33]	not found	99	0-4	2 researchers from study	No statement, transformation of Chew's list into numbers	yes	no	yes*	no	no	no	no	yes,
ABC	Anticholinergic Burden Classification	France /2006	Ancelin et al. [23]	not found	27	0-3	1 Pharmacologist, 1 Physician, 1 Biologist	Mean value	yes	no	yes	no	yes	yes	yes	no
ACB	Anticholinergic Cognitive Burden Scale	USA /2008	Boustani et al. [25, 27]	yes	88	0-3	Geriatricians, Geriatric pharmacists and psychiatrists and nurses, General physicians, Aging brain researcher	No statement	yes	no	yes	yes	no	no	yes	no
ACL (ALS)	Anticholinergic Load Scale	Australia / Thailand /2011	Sittironnarit et al.[42]	not found	49	0-3	2 psychiatrists, 1 clinical pharmacologists,1 geriatricians	Median value	yes	no	yes	no	yes*	yes*	yes*	yes
ADS	Anticholinergic Drug Scale	USA /2002/06	Carnahan et al. [29, 30]	yes	117	0-3	3 psychiatric pharmacists	Consensus formed	yes	no	yes	yes	no	no	no	yes
AEC	Anticholinergic Effect on Cognition	UK /2017	Bishara et al. [24]	not found	165	0-3	2 Researchers from study	Clear scoring rule, if discordant cross-checked by two independent authors if	no	no	yes	yes	no	no	yes	no
AIS	Anticholinergic Impregnation Scale	France /2017	Briet et al. [26]	not found	128	1-3	3 Clinical pharmacists , 2 Psychiatrists	Median value	yes	yes*	yes*	yes*	yes*	yes*	yes*	yes
ARS	Anticholinergic Risk Scale	USA /2008	Rudolph et al. [41]	not found	49	0-3	1 geriatrician, 2 geropharmacists	Median value	yes	no	yes, pKi Database	yes	no	no	no	no
ATS	Anticholinergic Toxicity Score	USA /2016	Xu et al. [44]	not found	25	0-5	No statement	Not applicable	no	no	yes, computational	no	no	no	no	no
BAADS	Brazilian Anticholinergic Activity Drug Scale	Brazil /2019	Nery et al. [40]	not found	125	1-3	No statement	No statement	yes*	yes*	yes*	yes*	yes*	yes*	yes*	yes
CABS	Cancelli's ACH Burden Scale	Italy /2008	Cancelli et al. [28]	not found	17	0-3	3 Neurologists	No statement	yes	yes	yes	yes	no	yes	no	no
Chew	Chew's Scale	USA /2008	Chew et al. [31]	not found	22	0, 0/+, + = 0.5-5 pmol/mL, ++ = 5-15 pmol/mL, +++ >15 pmol/mL	No statement	Not applicable	no	yes	yes	no	no	no	no	no
CI, PI	Clinical Index, Pharmacological Index	USA /2004	Minzenberg et al. [39]	yes	28	No borders fixed, relative values	10 psychiatrists	Mean value (PI, CI)	yes (CI)	no	yes (PI)	yes (CI)	no	no	no	no
CrAS	Clinician-rated Anticholinergic Scale	Canada /2001/ 08	Han et al. [34, 35]	yes	340	0-3	3 geriatric psychiatrists, (Update used 3 geriatricians)	Median value	yes	yes*	yes	yes	no	no	no	yes
DRS	Delirogenic Risk Scale	Germany /2015	Hefner et al. [36]	not found	106	1-4	No statement	In cases of discrepancies, Chew was preferred, followed by ADS and ABC.	yes*	yes	yes	yes	yes	yes	yes	yes
DS	Duran Scale	Ecuador /2013	Duran et al. [32]	not found	225	Low / High potency	No statement	Clear scoring rule	yes*	yes*	yes*	yes*	yes*	yes*	yes*	yes
GABS	German Anticholinergic Burden Scale	Germany /2018	Kiesel et al. [38]	not found	504	0-3	1 geriatrician, 2 clinical pharmacists	Clear scoring rule	yes	yes*	yes*	yes*	yes*	yes*	yes*	Yes
KABS	Korean Anticholinergic Activity Scale	Korea /2019	Jun et al. [37]	not found	494	0-3	Delphi process, 1 geriatrician, 1 internist, 1 neurologist, 1 psychiatrist, 3 clinical pharmacists	Clear scoring rule, if discordant Delphi process was applied	yes	yes*	yes*	yes*	yes*	yes*	yes*	yes
SCDL	Summer's Class of Drug List	USA /1978	Summers et al. [43]	not found	67	Class I-III	No statement	No statement	no	yes	yes	yes	no	no	no	no

yes * = criteria is mentioned in an ABS that is included in a prior published ABS (see Fig. 2)

Additionally, we were able to outline the relationship and dependencies of the identified ABS with one another. We found nine ABS that are not based on a prior published scale (Fig. 2). These are the ABC, ACB, AEC, ARS, ATS, CABS, Chew, SCDL and CI, PI.

**Fig. 2:**
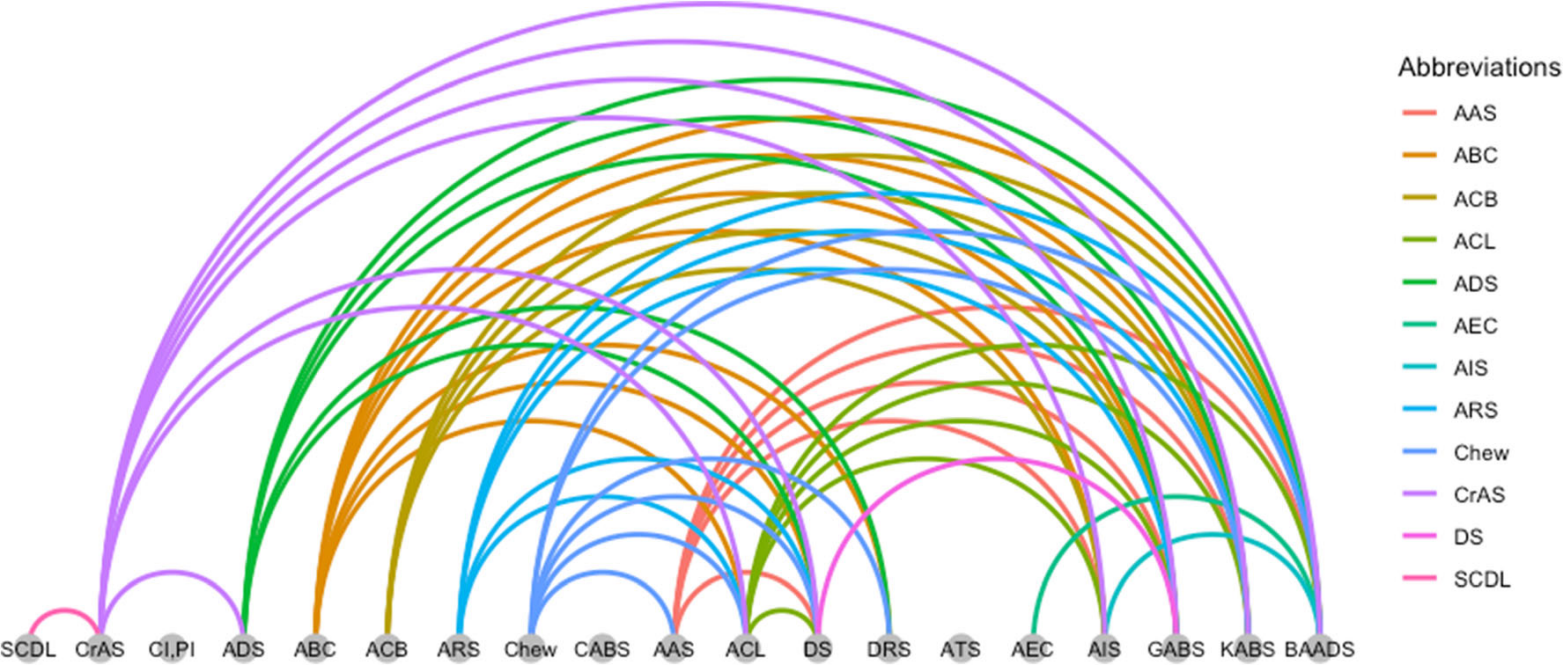
Relationship of the anticholinergic burden scales (ABS) sorted by the year of publication (from 1978 to 2019). Example of reading the figure: The SCDL is included in the CrAS or the CrAS is included in the ADS

**Fig. 3:**
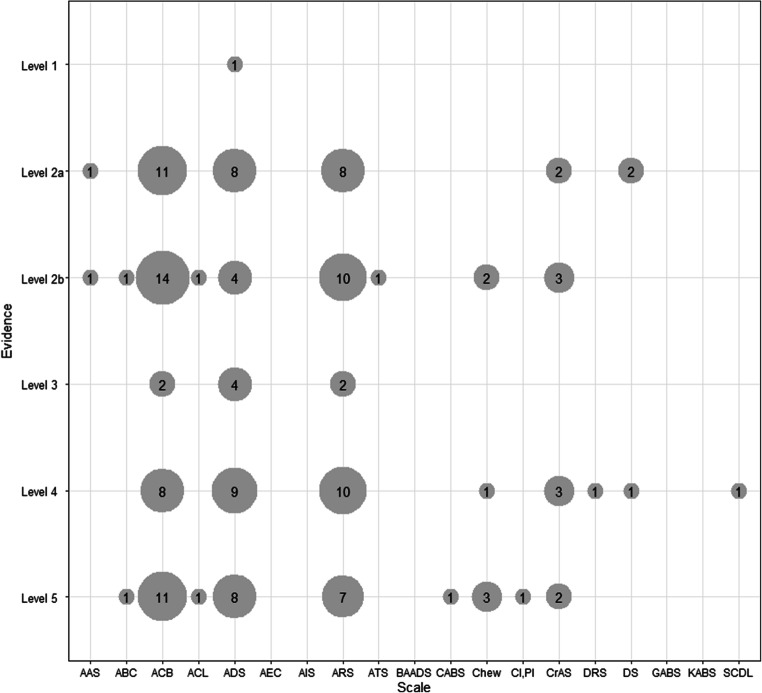
Count of validations per scale. Number of scale validations according to their level of evidence (total *n*=147). The bubble size is proportional to the number of validations per scale indicated as the numbers in the center of the bubble. Five ABS (AEC, AIS, BAADS, GABS and KABS) have not been validated yet

Our citation report analysis identified 104 validation studies [33–36, 39, 41, 44, 53–149] (Fig. 1, Appendix 2 and 6). We included one more study, when the search was updated [136]. Twenty reports compared more than one ABS leading to 147 evaluations with different clinical outcomes. The ACB, ADS and ARS are the scales mostly used while five ABS (AEC, AIS, BAADS, GABS and KABS) have not been validated yet (Fig. 3).

### The quality of the identified ABS and their validation studies

The ACB scale [25] and the GABS [38] reached with 75 %, the highest overall assessment percentage, while the SCDL [43] received the lowest with 11 % (Table 2). Focusing on domain 3 to 5 (“Rigor of development”, “Clarity of presentation” and “Applicability”), which were considered most important for clinical and research use, the ACB scale [25] reached the highest percentage in all three domains (62%, 89%, 72%) together with the GABS [38] for domain 5 (72%). The lowest percentage were achieved in domain 3 by the SCDL (16%) [43], domain 4 by the ABC scale (8%) [23] and domain 5 by the ABC [23], CABS [28], CI and PI [39] scales and ATS [44] (6%). In terms of clinical applicability, only the ACB, GABS and AEC provided an advice on how to apply the scale in clinical practice. The three scales agreed that a change in medication should be performed in a patient with a total ACB score > 2. Based on the quality assessment of the ABS with the adapted AGREE II tool, at least two out of three appraisers recommended all 19 ABS for use with modifications. The intraclass coefficient for absolute agreement was 0.89 with a 95%-CI ranging from 0.86–0.92, showing high agreement among the three appraisers [150].

**Table 2:** Systematic quality assessment of ABS by the adapted AGREE II tool

Domain	Domain 1: Scope and Purpose (%)	Domain 2: Stakeholder involvement (%)	Domain 3: Rigour of development (%)	Domain 4: Clarity of presentation (%)	Domain 5: Applicability (%)	Domain 6: Editorial independence (%)	Overall assessment (%)
AAS	59	28	31	28	17	65	33
ABC	52	36	25	8	6	70	25
ACB	67	64	62	89	72	89	75
ACL	61	56	38	28	17	4	28
ADS	65	56	51	42	33	94	61
AEC	70	64	49	78	56	87	72
AIS	65	50	25	42	39	48	25
ARS	70	75	58	67	33	50	67
ATS	74	31	40	39	6	87	36
BAADS	61	25	30	50	28	46	31
CABS	54	28	23	14	6	31	17
Chew	67	42	38	47	22	54	47
CI, PI	74	39	30	25	6	35	28
CrAS	57	44	54	31	22	61	53
DRS	67	28	31	44	17	11	33
DS	63	39	52	33	33	91	72
GABS	67	83	45	81	72	85	75
KABS	56	75	42	42	28	72	56
SCDL	39	8	16	25	11	6	11
Highest value	74	83	62	89	72	94	75
Lowest value	39	8	16	8	6	4	11
Median value	65	42	38	42	22	61	36

Numbers are scaled percentages for each domain 1–6 and the overall assessment calculated for interdomain comparison according to the manual

The 104 validation studies consisted of 1 RCT (good quality) [98], 74 cohort studies (50 good and 24 poor quality) [33–36, 41, 44, 54, 55, 57, 59, 61–66, 70–75, 77, 78, 80, 81, 83–97, 100, 103–108, 112–115, 117, 119, 120, 123, 124, 129, 131–137, 139, 141–146, 148, 149], 9 case-control studies (6 good, 1 fair and 2 poor quality) [56, 67–69, 102, 110, 118, 130, 147] and 20 cross-sectional studies (2 good and 18 poor quality) [39, 53, 58, 60, 76, 79, 82, 99, 101, 109, 111, 116, 121, 122, 125–128, 138, 140] (Appendix 6). More than half of the studies were judged to be of good quality (60 out of 104). There was only 1 RCT by Kersten et al. validating the ADS.

### Impact of ABS on clinical outcomes

**Fig. 4:**
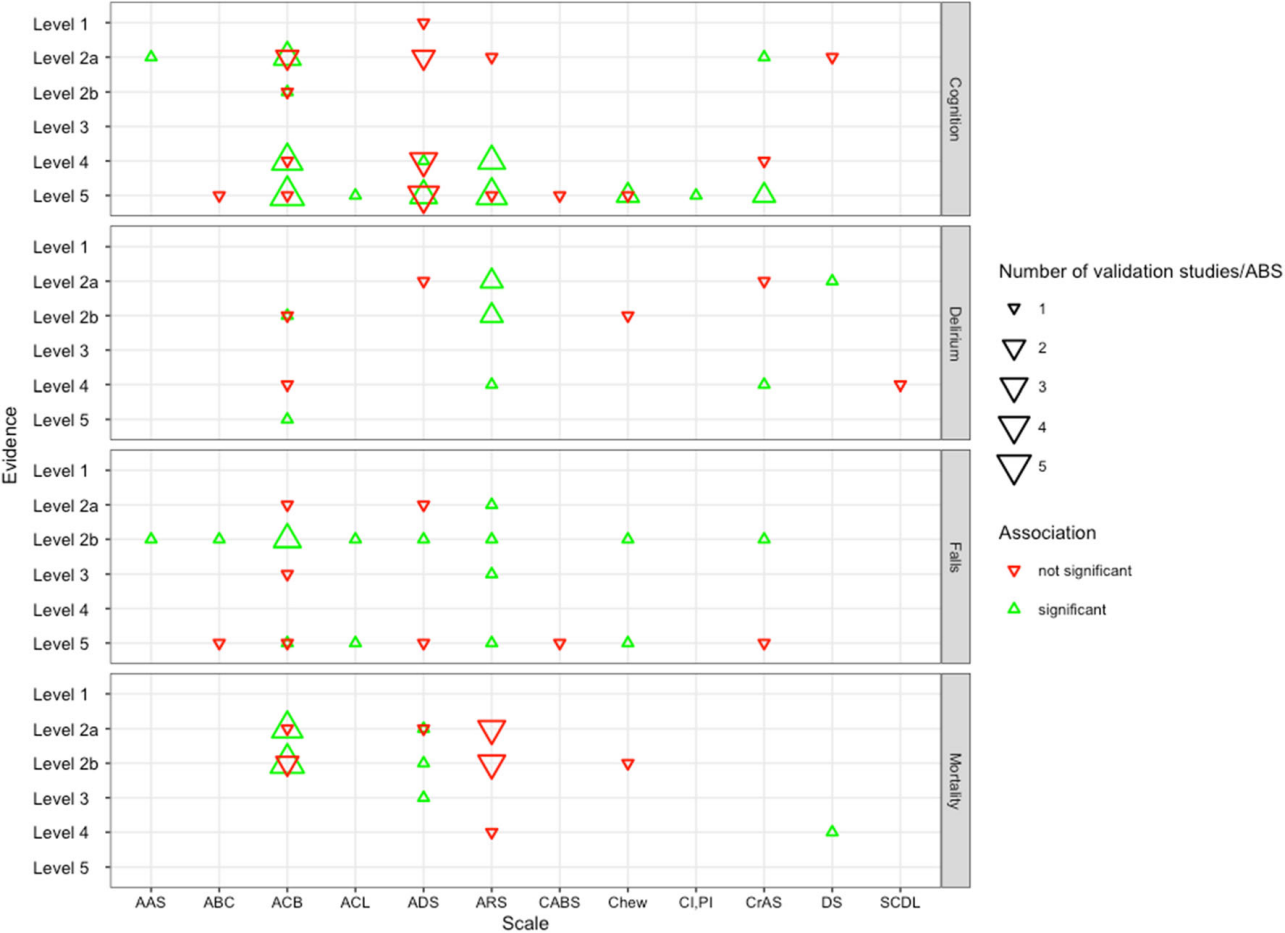
Found association of the validation studies with the most investigated clinical outcomes. Number of scale validations according to their evidence levels and grouped by the four most investigated clinical outcomes (total *n* = 118). The triangle size is proportional to the number of validations and an upward triangle means statistically significant association

From the 147 evaluations, 15 reported on delirium [35, 55, 62, 65, 74, 80, 96, 112, 126, 144, 149], 54 on cognition [33, 34, 39, 57–61, 64–66, 71, 73, 76, 79, 82, 83, 97–99, 106, 107, 109, 114, 121, 127, 128, 140, 145, 146, 148], 20 on mortality [55, 68, 72, 75, 80, 84, 85, 89, 100, 108, 113, 115, 117, 119, 120, 123, 132, 134, 137, 141] and 24 on falls [53, 87, 93, 112, 118, 121, 129, 131, 136, 147] (Fig. 4). As Fig. 4 demonstrates, the results are contradicting on all evidence levels. Yet, for all of these four clinical outcomes, the majority of studies show a positive association. In terms of study design, especially for falls and cognition, we identified many cross-sectional studies (cognition 27 out of 54, falls 9 out of 24) in contrast to delirium and mortality (delirium 1 out of 15, mortality 0 out of 20).

Other outcomes investigated in the studies can be found in (Appendix 6).

There is no study comparing the clinical performance of all published ABS in the measure of the cumulative ACH burden and its relationship with a clinical outcome. Only two out of the twenty reports comparing more than two ABS included up to eight ABS [121, 131]. These two reports consist of a cross-sectional study with poor quality (Level 5) [121] and a cohort study with good quality (Level 2b) [131]. Both of these studies investigated falls, where four ABS (ABC, ACB, ADS, CrAS) showed contradicting associations whereas three (ACL, ARS, Chew) agreed upon a positive association.

### Discussion

We identified 19 different published ABS and assessed their quality systematically. Among those ABS, 6 (GABS, KABS, ATS, DRS, AIS and AEC) have not been included in previous systematic reviews [6, 9–12]. Although at least two out of three appraisers recommended all scales for use with some modifications, their quality varied greatly. Firstly, although we were able to identify the source of evidence (e.g. BBB permeability, muscarinic receptor affinity), the clear explanation of the scoring rule and reproducibility of the scoring process was not always completely stated or left out. Secondly, the expert committee used for the development process varied in terms of number of experts and their field of expertise. Thirdly, most of the ABS were developed in the early 2000s, whereas only a few were published only recently. Hence, in the latter scores, there was not enough time to conduct a validation study. Our findings confirm as previously shown that no ABS can be considered a gold standard [52, 151].

Although more than half of the validation studies were of good quality and included sometimes an impressive sample size, many of them were cross-sectional studies, a design that is not optimal to assess adverse drug effects since ascertainment of exposure prior to outcome is not guaranteed. Classical RCTs, in which patients are randomly divided into treatment and placebo groups, are considered the highest level of evidence, but are not feasible here due to ethical issues. In this context, good quality cohort studies are best suited to assess a possible causal association. For example, the only identified RCT by Kersten et al. [98] conducted an intervention study to understand whether a reduced ADS score would improve cognition. This type of intervention study is very useful to investigate the impact of deprescribing, which can indirectly prove causal effects.

Despite the great number of validation studies, we were not able to measure the overall effect size for one of the four most investigated clinical outcomes. We encountered too much heterogeneity in terms of study design, study population and outcome measurement methods, rendering a meta-analysis impossible. So far, one study performed a meta-analysis for all-cause-mortality for the ACB scale and ARS with two studies per scale showing a tendency towards a positive association [152]. However, they also reported a significant heterogeneity in study population. Of note, it is worth mentioning that deleterious side effects of the CNS in the aging population are not solely linked to the ACH burden, but are probably multifactorial. ABS is one of the components to be considered when assessing the risk of CNS-related drug effects.

There are some limitations to this review. We did not search in grey literature to identify possible unpublished ABS or used other databases such as CINAHL or PsycINFO. However, we performed a rigorous search of the reference lists of the included studies. The quality assessment with the adapted AGREE II tool did not include the relationship of the scales with one another, which should be accounted for. Additionally, we selected only validation studies calculating the cumulative ACH burden and not studies using the medication lists to differentiate between users and non-users of ACH medication. Here we recommend the review by Mayer et al., where they distinguish the two ways of use [9]. To our understanding, calculating the cumulative ACH burden is the intended use of the ABS; otherwise, the scoring would be redundant. However, it is questionable whether the simple addition of the scores without considering the individual dosage and other factors such as the patient's renal function is the right approach to calculate the ACH burden.

Last, the combination of good and fair quality studies to assign evidence levels could have skewed the rating towards higher levels. However, only one study was rated as fair quality.

The strength of this review is its systematic approach applied from the search to the quality assessments. Though the original AGREE tool has been elaborated for guidelines and not for scales, it includes 13 quality dimensions and has been thoroughly evaluated [153]. Furthermore, the tool does not only include developmental aspects such as evidence basis but also clinical applicability and external review (here validation studies). This is the first review, which systematically assessed the quality of the ABS and their validation studies through adapted tools. Additionally, at least two researchers independently completed each step of the review process.

## Conclusion

We identified 19 published ABS with their validation studies and systematically assessed their quality using adapted tools. Despite differences in quality, all ABS were recommended for use with modifications. Most ABS have been validated; yet, validation studies for newer scales are lacking, and the evaluation of the association for the four most investigated clinical outcomes showed contradicting results. There is need for good quality cohort and intervention studies comparing multiple ABS to define the best scale for clinical use and to conduct a meta-analysis for the assessment of their clinical impact.

## Electronic supplementary material

ESM 1 (PDF 558 kb)

ESM 2 (PDF 533 kb)

ESM 3 (PDF 453 kb)

## Data Availability

Not applicable
