# Supplementary material for: Quality of anticholinergic burden scales and their impact on clinical outcomes: a systematic review
Source: Eur J Clin Pharmacol. 2020 Oct 3;77(2):147–62. doi: 10.1007/s00228-020-02994-x (PMC7803697; doi:10.1007/s00228-020-02994-x)
Supplement: Supplementary file 1 — (PDF 558 kb) [file 228_2020_2994_MOESM1_ESM.pdf]

Quality of anticholinergic burden scales and their impact on clinical outcomes - a systematic review, EJCP, Lisibach A et al, Corresponding author: Pr Chantal Csajka, Center for Research and Innovation in Clinical Pharmaceutical Sciences, Rue du Bugnon 17, 1005 Lausanne

Appendix 1: Search queries used in MEDLINE and EMBASE for the identification of all published ABS.

MEDLINE search query: ("anticholinergic"[Title] OR "anticholinergics"[Title]) AND ("scale"[Title] OR "risk scale"[Title] OR "scales"[Title] OR "properties"[Title] OR "score"[Title] OR "scores"[Title] OR "risk scales"[Title] OR "activities"[Title] OR "activity"[Title] OR "burden scale"[Title] OR "burden scales"[Title] OR "load"[Title] OR "burden"[Title] OR "effect"[Title] OR "effects"[Title]) AND "humans"[MeSH Terms] AND (French [lang] OR German[lang] OR English[lang])

EMBASE search query: (anticholinergic:ti OR anticholinergics:ti) AND (scale:ti OR 'risk scale':ti OR scales:ti OR properties:ti OR score:ti OR scores:ti OR 'risk scales':ti OR activities:ti OR activity:ti OR 'burden scale':ti OR 'burden scales':ti OR load:ti OR burden:ti OR effect:ti OR effects:ti) AND ([english]/lim OR [french]/lim OR [german]/lim) AND [humans]/lim

Appendix 2: Detailed PRISMA flowchart for the identification of all validation studies for the identified ABS.

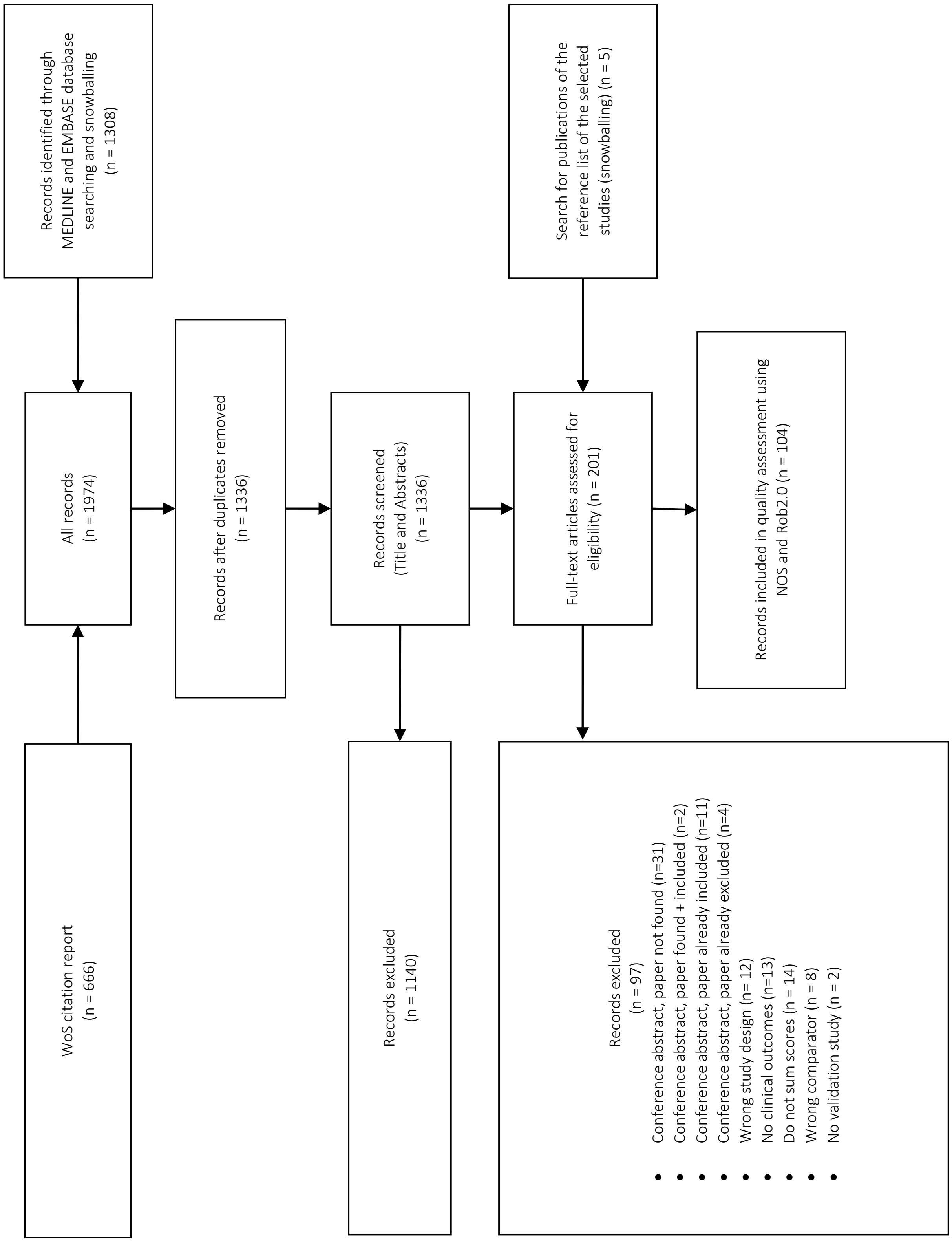

Appendix 3: The adapted AGREE II tool to assess the quality of the identified ABS.

There are 6 domains, every single item (numbered) below is graded from 1 to 7 by each researcher.

score 1 = strongly disagree (no information relevant on the respective item/ if it's reported very poorly)

score 2 - 6 = reporting doesn't meet the full criteria (score increases as more considerations are addressed

score 7 = strongly agree (in case reporting quality is exceptional, all criteria & considerations are met)

For the scoring, the numbered items in the LEFT column are the topics to be rated with signaling *questions* below.

The points with boxes in the column in the RIGHT column assist the scoring and could be identified.

CHECKLIST ITEM AND DESCRIPTION	REPORTING CRITERIA	Grade
**BASIC INFORMATION OF THE SCALE**		
**TITLE OF THE PUBLICATION**		
**YEAR OF PUBLICATION**		
**COUNTRY OF DEVELOPED SCALE**		
**ABBREVIATION OF SCALE**		
DOMAIN 1: SCOPE AND PURPOSE (Total: max. 21 P)		
**1. OBJECTIVES**<br><br>*Report the overall objective(s) of the paper. The expected health benefits from the developed scale are to be specific to the clinical problem/ health topic. Additionally: is it well written, clear and concise.*	☐ Health intent(s) (i.e., prevention, screening, diagnosis, treatment, etc.)<br>☐ Expected benefit(s) or outcome(s)<br>☐ Target(s) (e.g., patient population, society)	
**2. QUESTIONS**<br><br>*Report the health question(s) covered by the work, particularly for the key recommendations. Additionally: is it well written, clear and concise?*	☐ Target population<br>☐ Intervention(s) or exposure(s)<br>☐ Comparisons (if appropriate)<br>☐ Outcome(s)<br>☐ Health care setting or context	
**3. POPULATION**<br><br>*Describe the population (i.e., patients, public, etc.) to whom the scale is meant to apply. Additionally: is it well written, clear and concise.*	☐ Target population, sex and age<br>☐ Clinical condition (if relevant)<br>☐ Severity/stage of disease (if relevant)<br>☐ Comorbidities (if relevant)<br>☐ Excluded populations (if relevant)	

DOMAIN 2: STAKEHOLDER INVOLVEMENT (Total: max. 14 P)		
**4. GROUP MEMBERSHIP**<br>*(This may include members of the steering group, the research team involved in selecting and reviewing/rating the evidence and individuals involved in formulating the final recommendations.)*<br>*Do they report all individuals who were involved in the development process (expert panel)?*<br>*Do they mention them by:*<br>*Is there a minimum of 2 different researchers with two different backgrounds (e.g physician and clinical pharmacists)?*	☐ Yes<br>☐ No<br>☐ Not mentioned<br>Name, by disciple /content expertise, institution, geographical location, role in the scale development group<br>☐ Yes<br>☐ No<br>Expert panel number: _______<br>*Expert panel:*<br>☐ General physician<br>☐ Geriatric physician<br>☐ Clinical pharmacist<br>☐ Nurse<br>☐ Researcher<br>☐ Biologist<br>☐ Other *<br>* Other:___________	
**5. TARGET USERS**<br>*Report the target (or intended) users of the scale. Additionally: is it well written, clear and concise.*<br>*Is it well stated who is intended to use the scale? (specify, e.g. clinical pharmacists, physicians, nurses, patients)*<br>*Does it also mention how to be used by the target audience?*	☐ Yes<br>☐ No<br>☐ Yes<br>☐ No	
DOMAIN 3: RIGOUR OF DEVELOPMENT (Total: max. 63 P, Item 7 + 11 count double)		
**6. SEARCH METHODS**<br>*Do they report details of the strategy used to search for evidence for anticholinergic activity of a certain substance?*<br>*If yes, what is reported?*<br>*Additionally: Is the search relevant and appropriate to answer the health question.*	☐ Yes<br>☐ No<br>☐ Named electronic database(s) or evidence source(s) where the search was performed (e.g., MEDLINE, EMBASE, PsychINFO, CINAHL)<br>☐ Time periods searched (e.g., January 1, 2004 to March 31, 2008)	

Is there enough information provided for anyone to replicate the search?	☐ Search terms used (e.g., text words, indexing terms, subheadings)<br><br>☐ Full search strategy included (e.g., possibly located in appendix)<br><br>☐ Other literature* e.g. Martindale, Compendium<br>Specify other Literature*:__________________	
**7. EVIDENCE SELECTION CRITERIA**<br><br>*Report the criteria used to select (i.e., include and exclude) the evidence. Provide rationale, where appropriate.*<br><br>*(Each criterion included goes one point up the scale from 1 to 7)*	☐ Serum anticholinergic activity<br><br>☐ Pharmacokinetic / substance properties<br><br>☐ Anticholinergic side effects<br><br>☐ Blood-brain-barrier permeability of substance<br><br>☐ Taking dosage into account<br><br>☐ Route of administration was considered<br><br>☐ Clinical expert opinions<br><br>☐ Scale is based on previous published scale (includes reviews as well, e.g. Durán)	
**8. STRENGTHS & LIMITATIONS OF THE EVIDENCE**<br><br>*Describe the strengths/limitations of the evidence. Consider from the perspective of the individual studies and the body of evidence aggregated across all the studies. Statements highlighting the strengths/limitations of the evidence should be provided. This ought to include explicit descriptions - using informal or formal tools/methods - to assess and describe the risk of bias for individual studies and/or for specific outcomes and/or explicit commentary of the body of evidence aggregated across all studies. This may be presented in different ways, e.g: tables commenting on different quality domains; the application of a formal instrument or strategy; or descriptions in the text.*	☐ Several sources (e.g. in vitro and in vivo)<br><br>☐ Also clinical data (ADR)<br><br>☐ Quality assessment of the studies/data<br><br>☐ Consistency of results across studies/data<br><br>☐ Study design included in body of evidence<br><br>☐ Number of drugs that were evaluated<br><br>☐ Are all relevant drug classes included?<br><br>☐ Language: was the evidence not limited by the language?	
**9. FORMULATION OF RECOMMENDATIONS**<br><br>*Describe the methods used to formulate the recommendations and how final decisions were reached. Specify any areas of disagreement and the methods used to resolve them.*<br><br>*Was a clear scoring rule developed?*	☐ Yes<br><br>☐ No	

Do they reason their scoring?	☐ Yes<br>☐ No	
Scoring was performed by 2 or more independent researchers?	☐ Yes<br>☐ No	
Were there no discrepancies?	☐ Yes<br>☐ No	
If yes, do they mention how they resolved them?	☐ Yes<br>☐ No	
**10. LINK BETWEEN RECOMMENDATIONS AND EVIDENCE**<br>*Describe the explicit link between the recommendations and the evidence on which they are based.*	☐ Is there a clear link between the recommendation (e.g. classification) of a drug and the evidence? Is the classification reproducible?	
**11. VALIDATION OF SCALE**<br>*Has the scale been validated?*	☐ Yes<br>☐ No	
Were the studied primary and secondary outcomes appropriate?	☐ Yes<br>☐ No	
What was the studied outcome of the external review? Was it clinical?	______________________________________	
**12. UPDATING PROCEDURE**<br>*Describe the procedure for updating the work.*<br>*Has there been an update since the development of the scale?*<br>*If yes, when? (Was it provided by the authors?)*	☐ Yes *<br>☐ No<br>* Update: ____________________	

<table>
<tr><td colspan="3">DOMAIN 4: CLARITY OF PRESENTATION (Total: max. 14 P)</td></tr>
<tr><td>13. SPECIFIC AND UNAMBIGUOUS RECOMMENDATIONS

Describe which options are appropriate in which situations and in which population groups, as informed by the body of evidence.</td><td>☐ A statement of the recommended action

☐ Intent or purpose of the recommended action (e.g., to improve quality of life, to decrease side effects)

☐ Relevant population (e.g., patients, public)

☐ Caveats or qualifying statements, if relevant (e.g., patients or conditions to whom the recommendations would not apply)

☐ If there is uncertainty about the best care option(s), the uncertainty should be stated.</td><td></td></tr>
<tr><td>14. IDENTIFIABLE KEY RECOMMENDATIONS

Present the key recommendations so that they are easy to identify.

Is a full list of scored drugs available?

It is clearly described, how to use the scale? (clinical or research practice)</td><td>☐ Yes
☐ No

☐ Yes
☐ No</td><td></td></tr>
<tr><td colspan="3">DOMAIN 5: APPLICABILITY (Total: max. 7 P)</td></tr>
<tr><td>15. IMPLEMENTATION ADVICE/TOOLS

Provide advice and/or tools on how the recommendations can be applied in practice.</td><td>☐ Additional materials to support the implementation of the scale in practice.

o Web calculator of score
o List with anticholinergic drugs provided
o Links to how-to manuals
o Solutions linked to barrier analysis (Item 18)
o Tools to capitalize on facilitators (Item 18)</td><td></td></tr>
<tr><td colspan="3">DOMAIN 6: EDITORIAL INDEPENDENCE (Total: max. 21 P)</td></tr>
<tr><td>16. FUNDING BODY

Report the funding body's influence on the content of the scale.</td><td>☐ The name of the funding body or source of funding (or explicit statement of no funding)

☐ A statement that the funding body did not influence the content of the scale</td><td></td></tr>
<tr><td>17. COMPETING INTERESTS

Provide an explicit statement that all group members have declared whether they have any competing interests. Have there been any competing interests?</td><td>☐ Yes
☐ No</td><td></td></tr>
</table>

**18. SUGGESTIONS FOR FURTHER RESEARCH**<br><br>*Do they suggest further research?*<br><br>*Or is there profound explanation why such research isn't required currently?*	☐ Yes<br>☐ No *<br>* Explanation:______________________<br>___________________________________	
DOMAIN 7: OVERALL SCALE ASSESSMENT (Total: max. 14 P)		
**19. RATING OF THE OVERALL QUALITY OF THE SCALE**<br><br>*Rate the scale in a total overview from 1 (lowest possible quality) to 7 (highest possible quality).*	☐ It is of poorest quality (1)<br>☐ The quality should be improved in certain aspects (2-6)<br>☐ Quality is exceptional (7)	
**20. RECOMMENDATION FOR USE**<br><br>*Decide whether the scale could be recommended for good results, or not.*	☐ Yes<br>☐ Yes, with modifications<br>☐ No	
**Comments:**		

From: Brouwers MC, Kerkvliet K, Spithoff K, on behalf of the AGREE Next Steps Consortium. The AGREE Reporting Checklist: a tool to improve reporting of clinical practice guidelines. *BMJ* 2016;352:i1152. doi: 10.1136/bmj.i1152.

For more information about the AGREE Reporting Checklist, please visit the AGREE Enterprise website at http://www.agreetrust.org.

Appendix 4: The slightly adapted NOS tools for case-control, cohort studies and cross-sectional studies used for the quality assessment of each validation studies. The dot indicates a star.

**Case-control studies**	**Cohort / Cross-sectional studies**
**Selection**	**Selection**
1) Is the case definition adequate?	***1) Representativeness of the exposed cohort***
a) yes, with independent variables ●	a) truly representative of the average … (describe) in the community ●
b) yes, e.g. record linkage or based on self-reports	b) somewhat representative of the average … (describe) in the community ●
c) no description	c) selected group of users eg nurses, volunteers
	d) no description of the derivation of the cohort
2) Representativeness of the cases	***2) Selection of the non-exposed cohort***
a) consecutive or obviously representative series of cases ●	a) drawn from the same community as the exposed cohort ●
b) potential for selection bias or not stated	b) drawn from a different source
	c) no description of the derivation of the non-exposed cohort
3) Selection of controls	***3) Ascertainment of exposure / Measurement method of exposure***
a) community controls ● (same population)	a) secure record (eg surgical records) ● / validated measurement tool ●
b) hospital controls	b) structured interview ● / some measurement tool ●
c) no description	c) written self-report
	d) no description
4) Definition of controls	***4) Demonstration that outcome of interest was not present at start of study or baseline measurement / Always "no" in cross-setional***
a) no history of disease (endpoint) ●	a) yes ●
b) no description of source	b) no
**Comparability**	**Comparability**
1) Comparability of cases and controls on the basis of design or analysis	***1) Comparability of cohorts on the basis of the design or analysis***
a) study controls for… (select the most important factor) ●<br>Identified most important factor: (write down for each study)	a) study controls for… (select the most important factor) ●<br>Identified most important factor: (write down for each study)
b) study controls for any additional factor (could be a second most important factor) ●<br>Identified factor: (write down for each study)	b) study controls for any additional factor (could be a second most important factor) ●<br>Identified factor: (write down for each study)

Exposure	Outcome
1) Ascertainment of exposure	***1) Ascertainment of outcome***
a) secure records (e.g surgical records) ●	a) independent blind assessment ●
b) structured interview where blind to case/ control status ●	b) record linkage ●
c) interview not blinded to case / control status	c) self-report
d) written self-report or medical record only<br>e) no description	d) no description
2) Same method of ascertainment for cases and controls	***2) Was follow-up long enough for outcomes to occur / Always "no" in cross-setional***
a) yes ●	a) yes ●
b) no	Selected adequate time of follow-up
3) Missing data	***3) Adequacy of follow up of cohorts / Missing data for cross-sectional***
a) described how much missing data and how they handled it ●	a) complete follow up - all subjects accounted for ● / no missing data ●
b) mention missing data but no further explanation	b) subjects lost to follow up unlikely to introduce bias: < 10 (20%)% (Oxford Center of EBM) ● / described how much missing data and how they handled it ●<br><br>Adequate number: *If <20% of subjects were lost to follow-up but the difference between the groups is large consider downgrading to c, especially if no reason is given*
c) no description	c) follow-up rate < 80 % and not description of those lost / mention missing data but no further explanation
	d) no statement / no description

Appendix 5: AHRQ standards conversion rules for the quality assessed by the NOS and Rob2.0.

**Quality assessed by the NOS for cohort, case control and cross-sectional studies:**
**Good quality:** 3 or 4 stars in selection domain AND 1 or 2 stars in comparability domain AND 2 or 3 stars in outcome/exposure domain
**Fair quality:** 2 stars in selection domain AND 1 or 2 stars in comparability domain AND 2 or 3 stars in outcome/exposure domain
**Poor quality:** 0 or 1 star in selection domain OR 0 stars in comparability domain OR 0 or 1 stars in outcome/exposure domain

**Quality assessed by the Rob2.0 for RCT studies:**
**Good quality:** low risk of bias for each domain and all criteria met
**Fair quality:** high risk of bias for one domain or two criteria unclear risk of bias
**Poor quality:** two or more criteria listed as high or unclear risk of bias
