# Supplementary material for: Quality of anticholinergic burden scales and their impact on clinical outcomes: a systematic review
Source: Eur J Clin Pharmacol. 2020 Oct 3;77(2):147–62. doi: 10.1007/s00228-020-02994-x (PMC7803697; doi:10.1007/s00228-020-02994-x)
Supplement: Supplementary file 3 — (PDF 453 kb) [file 228_2020_2994_MOESM3_ESM.pdf]

Quality of anticholinergic burden scales and their impact on clinical outcomes - a systematic review, EJCP, Lisibach A et al, Corresponding author: Pr Chantal Csajka ,Center for Research and Innovation in Clinical Pharmaceutical Sciences, Rue du Bugnon 17, 1005 Lausanne

Appendix 6b: Identified validation studies (total n=104).

Number of compared ABS	ABS	Study design	Year	Author	Title	Study population	Clinical outcome	Evidence level (adapted EBM Oxford)	Quality (NOS, RoB 2.0)	Delirium	Cognition	Mortality	Falls
1	ADS	RCT	2013	Kersten et al.	Cognitive effects of reducing anticholinergic drug burden in a frail elderly population: a randomized controlled trial	Nursing home residents,<br>n = 87 with a mean age of 85 years	Cognitive function<br>Mouth dryness	1	Good	0	1	0	0
2	CrAS, SCDL	Cohort study	2001	Han et al.	Use of Medications with Anticholinergic Effect Predicts Clinical Severity of Delirium Symptoms in Older Medical Inpatients	Inpatients with delirium,<br>n = 278 with a mean age of 83.4 ± 7.3 years	Change in severity of delirium symptoms<br>Dementia diagnosis	4	Poor	1	0	0	0
1	CrAS	Cohort study	2008	Han et al.	Cumulative anticholinergic exposure is associated with poor memory and executive function in older men	Community-dwelling men,<br>n = 544 with a mean age of 74.4 ± 5.2 years	Short term memory (HVRT)<br>Executive function (IADL)	2a	Good	0	1	0	0
1	ARS	Cohort study	2008	Rudolph et al.	The Anticholinergic Risk Scale and Anticholinergic Adverse Effects in Older Persons	Inpatient of 2 cohorts<br>retrospective cohort:<br>n = 132 with a mean age of 78.7 ± 5.3 years<br>prospective cohort:<br>n = 117 male with a mean age of 71.5 ± 11.6 years	Central and peripheral anticholinergic adverse effects (c: falls, dizziness, confusion, p: dry mouth, dry eye, constipation)	4	Poor	0	0	0	0
1	CrAS	Cohort study (reanalysis on a RCT)	2009	Agar et al.	Changes in anticholinergic load from regular prescribed medications in palliative care as death approaches	Palliative care patients,<br>n = 461 with a mean age of 71 ± 12 years	Quality of life (McCill's Quality of life index)<br>Functional outcome (Karnofsky performance scale)	4	Poor	0	0	0	0
1	CrAS	Cohort study	2009	Juliebo et al.	Risk Factors for Preoperative and Postoperative Delirium in Elderly Patients with Hip Fracture	Orthopedic inpatients with hip fracture,<br>n = 364 with a mean age of 84 ± 4 years	Delirium (CAM, pre- and postoperatively)	2a	Good	1	0	0	0
1	ADS	Cohort study	2009	Low et al.	Use of medications with anticholinergic properties and cognitive function in a young-old community sample	Community-dwelling patients,<br>n = 2058 with a mean age of 62.5 ± 1.5 years	Mild cognitive impairment	2a	Good	0	1	0	0
1	ACB	Cohort study	2010	Campbell et al.	Use of anticholinergics and the risk of cognitive impairment in an African American population	Community-dwelling African American,<br>n = 1652 with a mean age of 81.8 ± 5.3 years	Cognitive Impairment	2a	Good	0	1	0	0
1	CrAS	Cohort study	2010	Clark et al.	The impact of opioids, anticholinergic medications and disease progression on the prescription of laxatives in hospitalized palliative care patients: a retrospective analysis	Palliative care,<br>n = 211 with a mean age of 72.3 ± 14.72 years	Prescription of laxatives	2b	Good	0	0	0	0
1	AAS	Cohort study	2010	Ehrt et al.	Use of drugs with anticholinergic effect and impact on cognition in Parkinson's disease: a cohort study	Community-based PD patients,<br>n = 235 with a mean age of 74.7 ± 8.4 years	Cognitive function (MMSE)	2a	Good	0	1	0	0
1	Chew	Cohort study	2010	Jessen et al.	Anticholinergic drug use and risk for dementia: target for dementia prevention	Outpatients,<br>n = 2605 and aged > 70 years	Dementia	4	Poor	0	0	0	0
1	ACB	Cohort study	2011	Campbell et al.	Association between prescribing of anticholinergic medications and incident delirium: a cohort study	Hospitalized patients with cognitive impairment,<br>n = 147 aged ≥ 65 years	Delirium (CAM)	4	Poor	1	0	0	0
1	ACB	Cohort study	2011	Fox et al.	The impact of anticholinergic burden in Alzheimer's Dementia-the Laser-AD study	Nursing & residual homes, in- and outpatients with Alzheimer dementia (AD),<br>n = 224 with a mean age of 81 ± 7.4 years,	Cognitive function<br>(MMSE, SIB, ADAS-COG score)	2a	Good	0	1	0	0
1	ACB	Cohort study (multicenter)	2011	Fox et al.	Anticholinergic medication use and cognitive impairment in the older population: the medical research council cognitive function and ageing study	In- and outpatients with complete MMSE score and medication, n = 12'250 aged ≥ 65 years	Cognitive function (MMSE)<br>Mortality	2a	Good	0	1	1	0
1	ARS	Cohort study (multicenter)	2011	Kumpula et al.	Anticholinergic Drug Use and Mortality Among Residents of Long-Term Care Facilities: A Prospective Cohort Study	Hospital and long-term care, n = 1004<br>ARS 0: n = 455, with a mean age of 83.0 ± 10.0 years<br>ARS 1-2: n = 363, with a mean age of 80.5 ± 11.0 years<br>ARS ≥ 3: n = 186, with a mean age of 78.7 ± 12.3 years	Mortality	2a	Good	0	0	1	0
1	ARS	Cohort study (multicenter)	2011	Lowry et al.	Associations Between the Anticholinergic Risk Scale Score and Physical Function: Potential Implications for Adverse Outcomes in Older Hospitalized Patients	Inpatients,<br>n = 362 with a mean age of 83.6 ± 6.6 years	Physical function (BI)<br>Mortality<br>LOS	2a	Good	0	0	1	0
1	CrAS	Cohort study	2011	Sheehan et sl.	A Retrospective Analysis of Primary Diagnosis, Comorbidities, Anticholinergic Load, and Other Factors on Treatment for Noisy Respiratory Secretions at the End of Life	Inpatients dying in the palliative care unit,<br>n = 199 with a mean age of 70.5 ± 14.8 years	Need for treatment of noisy respiratory excretions (NRS)	2b	Good	0	0	0	0
2	ADS, ARS	Cohort study	2012	Gouraud-Tanguy et al.	Analysis of iatrogenic risk related to anticholinergic effects using two scales in acute geriatric inpatient unit	Patients from the geriatric care unit,<br>n = 1379 with a mean age of 85 ± 6 years	Total anticholinergic side effects<br>Peripheral anticholinergic side effects<br>Central anticholinergic side effects	4	Poor	0	0	0	0
1	ARS	Cohort study	2012	Koshoedo et al.	Anticholinergic Drugs and Functional Outcomes in Older Patients Undergoing Orthopaedic Rehabilitation	Patients from a rehabilitation unit,<br>n = 117 with a mean age of 79 ± 7 years,<br>with ACH: n = 38 with a mean age of 77 ± 7 years and without ACH: n = 79 with a mean age of 80 ± 6 years	Functional outcome (BI)<br>LOS	2a	Good	0	0	0	0
1	ACB	Cohort study	2013	Koyama et al.	Ten-year trajectory of potentially inappropriate medications in very old women: importance of cognitive status	Community-dwelling women,<br>n = 1484, at baseline with n = 358 users with a mean age of 78 ± 3.1 years and n = 1115 non-users with a mean age of 78 ± 3.2 years	Cognitive function (MCI)<br>Dementia	4	Poor	0	1	0	0

Quality of anticholinergic burden scales and their impact on clinical outcomes - a systematic review, EJCP, Lisibach A et al, Corresponding author: Pr Chantal Csajka ,Center for Research and Innovation in Clinical Pharmaceutical Sciences, Rue du Bugnon 17, 1005 Lausanne

Appendix 6b: Identified validation studies (total n=104).

Number of compared ABS	ABS	Study design	Year	Author	Title	Study population	Clinical outcome	Evidence level (adapted EBM Oxford)	Quality (NOS, RoB 2.0)	Delirium	Cognition	Mortality	Falls
3	ACB, ADS, ARS	Cohort study	2013	Mangoni et al.	Measures of Anticholinergic Drug Exposure, Serum Anticholinergic Activity, and All-cause Post discharge Mortality in Older Hospitalized Patients with Hip Fractures	Patients with hip fractures and planned surgery,<br>n = 71 with a mean age of 84 ± 6 years	Postoperative complications<br>LOS<br>3 month all-cause mortality<br>1 year all-cause mortality	2a	Good	0	0	1	0
1	CrAS	Cohort study	2013	Yeh et al.	Potential benefits of reducing medication-related anticholinergic burden for demented older adults: A prospective cohort study	Veteran home demented residents,<br>n = 53 with a mean age of 83.4 years	Cognitive function (MMSE)<br>Functional outcome (BI)	4	Poor	0	1	0	0
1	ARS	Cohort study	2014	Dispennette et al.	Drug Burden Index score and anticholinergic risk scale as predictors of readmission to the hospital	Inpatients, n = 229 with a mean age of 78 years	Risk of hospital readmission	4	Poor	0	0	0	0
2	ADS, ARS	Cohort study	2014	Kalisch et al.	Multiple Anticholinergic Medication Use and Risk of Hospital Admission for Confusion or Dementia	Australian veterans,<br>n = 36'015 with a mean age of 82.9 ± 6.8 years	Risk of hospitalization for confusion or dementia	2b	Good	0	0	0	0
3	ACB, ADS, ARS	Cohort study	2014	Kashyap et al.	Methodological Challenges in Determining Longitudinal Associations Between Anticholinergic Drug Use and Incident Cognitive Decline	Outpatient clinics without dementia or depression,<br>n = 102 with a mean age of 71.9 ± 7.3 years	Cognitive function (MMSE, GDP)	4	Poor	0	1	0	0
1	ACB	Cohort study (multicenter)	2014	Kidd et al.	The relationship between total anticholinergic burden (ACB) and early in-patient hospital mortality and length of stay in the oldest old aged 90 years and over admitted with an acute illness	Inpatients, n = 419 with a median age of 92.9 years,<br>ACB 0: n = 163 with a median age of 93.4 years<br>ACB 1: n = 142 with a median age of 92.9 years<br>ACB ≥ 2: n = 114 with a median age of 92.5 years	Mortality<br>LOS	2b	Good	0	0	1	0
1	ACB	Cohort study (multicenter)	2014	Koyama et al.	Long-term cognitive and functional effects of potentially inappropriate medications in older women	Community-dwelling women,<br>n = 1429 with a mean age of 83.2 ± 3.3 years	Functional outcome (IADL)<br>Cognitive function (MMSE)	4	Poor	0	1	0	0
1	ARS	Cohort study (multicenter)	2014	Landi et al.	Anticholinergic Drug Use and Negative Outcomes Among the Frail Elderly Population Living in a Nursing Home	Nursing homes residents,<br>n = 1490 with a median age of 83.56 years	Functional decline<br>Falls<br>Delirium	2a	Good	1	0	0	1
1	ARS	Cohort study	2014	Walter et al.	Perioperative Anticholinergic Medications and Risk of Catheterization After Urogynecologic Surgery	Female outpatients, n = 125,<br>ACH 0-7: n = 98 with a mean age of 56.0 ± 12.1 years<br>ACH ≥ 8: n = 27 with a mean age of 55.3 ± 11.2 years	Failed post-operative void trial	2b	Good	0	0	0	0
1	ARS	Cohort study	2014	Zimmerman et al.	Increasing anticholinergic burden and delirium in palliative care inpatients	Palliative inpatients,<br>n = 217 with a mean age of 72.9 ± 12.8 years	Delirium (Chart review)	4	Poor	1	0	0	0
1	ADS	Cohort study	2015	Block et al.	The interaction between medical burden and anticholinergic cognitive burden on neuropsychological function in a geriatric primary care sample	Primary care patients (outpatients),<br>n = 290 with a mean age of 72.76 ± 5.47 years	Neuropsychological functions (RBANS test)	4	Poor	0	1	0	0
1	ARS	Cohort study	2015	De La Cruz et al.	Impact of anticholinergic load on bladder function	Women undergoing urodynamics, n = 599,<br>low ACH: n = 440 with mean age of 57.2 ± 14 years<br>high ACH: n = 159 with mean age of 56.3 ± 12.9 years	Bladder function	4	Poor	0	0	0	0
1	ADS	Cohort study	2015	Gupte et al.	Impact of anticholinergic load of medications on the length of stay of cancer patients in hospice care	Cancer patients discharge from hospice care, selected by ICD-9 codes 140 - 239, n = 1801<br>ADS 0-2: n = 641, mean age of 72.38 ± 13.50 years<br>ADS 3-5: n = 637, mean age of 71.75 ± 13.83 years<br>ADS ≥ 6: n = 523, mean age of 70.42 ± 13.79 years	LOS / survival in hospice care (US)	2b	Good	0	0	0	0
1	ATS	Cohort study	2015	Hefner et al.	Rating The Delirogenic Potential of Drugs for Prediction of Side Effects in Elderly Psychiatric Inpatients	Psychiatric patients with TDM,<br>n = 69 with a mean age of 74.3 ± 5.7 years	Anticholinergic side effects (peripheral and central)	4	Poor	0	0	0	0
1	ARS	Cohort study	2015	Lu et al.	Effect of polypharmacy, potentially inappropriate medications and anticholinergic burden on clinical outcomes: a retrospective cohort study	Using the Taiwan's National Health Insurance Research Database (NHIRD),<br>n = 59'042 aged > 65 years	All-cause admission to hospital<br>Fracture-specific admission to hospital<br>Death	2b	Good	0	0	1	0
1	ACB	Cohort study	2015	Myint et al.	Total anticholinergic burden and risk of mortality and cardiovascular disease over 10 years in 21,636 middle-aged and older men and women of EPIC-Norfolk prospective population study	Community-dwelling patients without cancer,<br>n = 21'636<br>ACB 0: n = 17'317 with mean age of 57.9 ± 9.1 years<br>ACB 1: n = 2704 with mean age of 62.9 ± 8.8 years<br>ACB 2-3: n = 1324 with mean age of 62.2 ± 9.2 years<br>ACB > 3: n = 291 with mean age of 63.1 ± 8.9 years	All-cause mortality<br>Incident CVD	2b	Good	0	0	1	0
1	ACB	Cohort study	2015	Richardson et al.	Use of Medications with Anticholinergic Activity and Self-Reported Injurious Falls in Older Community-Dwelling Adults	Community-dwelling patients without dementia,<br>n = 2696 aged ≥ 65 years,<br>men (m) n = 1286 and women (w) n = 1410	Injurious falls (m/f)<br>Any falls (m/f)<br>Total number of falls (m/f)	2a	Good	0	0	0	1
8	ACB, ADS, ARS, ABC, ACL, Chew, CrAS, AAS	Cohort study	2015	Salahudeen et al.	Comparison of anticholinergic risk scales and associations with adverse health outcomes in older people	Outpatients,<br>n = 537'387 with a mean age of 74.7 ± 7.6 years	Hospital admissions<br>Falls-related hospitalizations<br>LOS<br>GP visits	2b	Good	0	0	0	1
2	ADS, ARS	Cohort study	2015	Wolters et al.	Anticholinergic Medication Use and Transition to Delirium in Critically Ill Patients: A Prospective Cohort Study	Critically ill inpatients,<br>n = 1112 with a mean age of 60 ± 16 years	Delirium onset (ICU-CAM)	2a	Good	1	0	0	0

Quality of anticholinergic burden scales and their impact on clinical outcomes - a systematic review, EJCP, Lisibach A et al, Corresponding author: Pr Chantal Csajka ,Center for Research and Innovation in Clinical Pharmaceutical Sciences, Rue du Bugnon 17, 1005 Lausanne

Appendix 6b: Identified validation studies (total n=104).

Number of compared ABS	ABS	Study design	Year	Author	Title	Study population	Clinical outcome	Evidence level (adapted EBM Oxford)	Quality (NOS, RoB 2.0)	Delirium	Cognition	Mortality	Falls
1	ADS	Cohort study	2015	Yarnall et al.	Anticholinergic Load: Is there a Cognitive Cost in Early Parkinson's Disease?	Community-dwelling and outpatients with Parkinson,<br>PD-ADS 0: n = 112 with a mean age of 68.6 ± 8.9 years<br>PD+ADS ≥ 1: n = 84 with a mean age of 69.7 ± 7.7	Mild cognitive impairment in Parkinson's disease	4	Poor	0	1	0	0
1	ACB	Cohort study	2016	Campbell et al.	Association of Anticholinergic Burden with Cognitive Impairment and Health Care Utilization Among a Diverse Ambulatory Older Adult Population	Community-dwelling patients,<br>n = 3344 aged ≥ 65 years	Cognitive impairment<br>Inpatient, ED and outpatient visit	2b	Good	0	1	0	0
1	ARS	Cohort study	2016	Crispo et al.	Associations between Anticholinergic Burden and Adverse Health Outcomes in Parkinson Disease	Patients with Parkinson disease,<br>n = 16'302 aged ≥ 40 years	Delirium<br>Fracture<br>30-day hospital revisits	2b	Good	1	0	0	0
1	ADS	Cohort study (reanalysis on a RCT)	2016	Hochman et al.	Anticholinergic Drug Burden in Noncancer Versus Cancer Patients Near the End of Life	Comparing patients with cancer n = 126 with a mean age of 79.1 ± 10.6 years and without cancer n = 118 with a mean age of 69.7 ± 10.5 years	Fatigue<br>Quality of life (QOL) (worse)<br>Drowsiness<br>Well-being	4	Poor	0	0	0	0
1	ARS	Cohort study	2016	Mangoni et al.	Heat Waves, Drugs with Anticholinergic Effects, and Outcomes in Older Hospitalized Adults	Hospitalized patients n = 307 in heat waves period with a median age 78 years, n = 1114 during non heat waves period with a median age 77 years	LOS<br>In-hospital mortality	2b	Good	0	0	1	0
1	ACB	Cohort study	2016	Vetrano et al.	Anticholinergic Medication Burden and 5-Year Risk of Hospitalization and Death in Nursing Home Elderly Residents With Coronary Artery Disease	Nursing home residents,<br>n = 3781 with a mean age of 83 ± 7 years	Hospitalization<br>Mortality	2b	Good	0	0	1	0
1	DS	Cohort study (multicenter)	2017	Burry et al.	Delirium and exposure to psychoactive medications in critically ill adults: A multi-centre observational study	Critically ill adults admitted to the ICU,<br>n = 520 aged ≥ 18 years	Delirium (ICDSC)	2a	Good	1	0	0	0
3	ACB, ADS, ARS	Cohort study	2017	Cossette et al.	Association Between Anticholinergic Drug Use and Health-Related Quality of Life in Community-Dwelling Older Adults	Community-dwelling patients free of disabilities in ADL and cognitive impairment,<br>n = 1793 with a mean age of 74.4 ± 4.2 years	Mental Component Summary (MCS)<br>Physical Component Summary (PCS)	4	Poor	0	1	0	0
1	ACB	Cohort study (multicenter)	2017	Cross et al.	Potentially Inappropriate Medication, Anticholinergic Burden, and Mortality in People Attending Memory Clinics	Community-dwelling patients with mild cognitive impairment or dementia of 9 memory clinics,<br>n = 964 with a mean age of 77.6 ± 7.4 years	Mortality	2a	Good	0	0	1	0
3	ACB, ARS, Chew	Cohort study	2017	Egberts et al.	Anticholinergic drug exposure is associated with delirium and post discharge institutionalization in acutely ill hospitalized older patients	Acutely ill, hospitalized patients (> 3 days),<br>n = 905 with a mean age of 81.0 ± 7.03 years	Delirium on admission<br>LOS<br>Post discharge institutionalization<br>In hospital mortality	2b	Good	1	0	1	0
2	ARS, DS	Cohort study (multicenter)	2017	Gutierrez et al.	Anticholinergic burden and health outcomes among older adults discharged from hospital: results from the CRIME study	Inpatients total,<br>n = 921 with mean age of 81.2 ± 7.4 years<br>For ARS:<br>ARS 0: n = 740 with a mean age of 80.9 ± 7.3 years<br>ARS 1: n = 132 with a mean age of 82.6 ± 72 years<br>ARS ≥ 2: n = 49 with a mean age of 81.1 ± 7.8 years<br>For Duran:<br>Duran 0: n = 625 with a mean age of 80.7 ± 7.3 years<br>Duran 1: n = 218 with a mean age of 82.0 ± 7.4 years<br>Duran ≥ 2: n = 78 with a mean age of 82.1 ± 7.1 years	Mortality within 1 year of discharge<br>Rehospitalization within 1 year of discharge	4	Poor	0	0	1	0
1	ADS	Cohort study (multicenter)	2017	Jean-Bart et al.	Exposure to anticholinergic and sedative medicines as indicators of high-risk prescriptions in the elderly	Inpatients,<br>n = 315 with a mean age of 86.6 ± 6.2 years	Risk of falls	2a	Good	0	0	0	1
1	ACB	Cohort study	2017	Naharci et al.	Effect of anticholinergic burden on the development of dementia in older adults with subjective cognitive decline	Patients with subjective cognitive decline,<br>n = 109 with a mean age 72.5 ± 63 years	Dementia	2b	Good	0	0	0	0
1	ADS	Cohort study	2017	Sarbacker et al.	Total anticholinergic burden and survival within a cohort of elderly Mexican Americans	Mexican Americans,<br>n = 1497 with a mean age of 74.56 years	Mortality	2b	Good	0	0	1	0
1	ADS	Cohort study (for part of outcomes)	2017	Sevilla-Sanchez et al.	Adverse drug events in patients with advanced chronic conditions who have a prognosis of limited life expectancy at hospital admission	Inpatients requiring palliative care,<br>n = 235 with a mean age of 86.8 ± 5.37 years	Survival	2a	Good	0	0	0	0
1	ADS	Cohort study	2017	Weglinski et al.	Prospective evaluation of mouth and eye dryness induced by antimuscarinic drugs used for neurogenic overactive bladder in 35 patients with multiple sclerosis	Community-dwelling patients with MS,<br>n = 35 with a mean age of 50.1 ± 10.2 years	Eye dryness (Xerophtalmia)<br>Mouth dryness (Xerostomia)	4	Poor	0	0	0	0
2	ACB, ARS	Cohort study	2017	Wen-Han Hsu et al.	Comparative Associations Between Measures of Anti-cholinergic Burden and Adverse Clinical Outcomes	Inpatients, n = 116'043 aged > 65 years	Emergency Department Visits<br>All-Cause Hospitalizations<br>Fracture-Specific Hospitalization<br>Incident dementia	2b	Good	0	0	0	0
1	ATS	Cohort study	2017	Xu et al.	Assessing and predicting drug-induced anticholinergic risks: an integrated computational approach	Patients, exposed n = 287'614 and unexposed n = 287'614, with a mean age of 37.97 ± 18.79 years	Anticholinergic ADE	2b	Good	0	0	0	0

Quality of anticholinergic burden scales and their impact on clinical outcomes - a systematic review, EJCP, Lisibach A et al, Corresponding author: Pr Chantal Csajka ,Center for Research and Innovation in Clinical Pharmaceutical Sciences, Rue du Bugnon 17, 1005 Lausanne

Appendix 6b: Identified validation studies (total n=104).

Number of compared ABS	ABS	Study design	Year	Author	Title	Study population	Clinical outcome	Evidence level (adapted EBM Oxford)	Quality (NOS, RoB 2.0)	Delirium	Cognition	Mortality	Falls
2	ACB, ARS	Cohort study	2018	Brombo et al.	Association of Anticholinergic Drug Burden with Cognitive and Functional Decline Over Time in Older Inpatients: Results from the CRIME Project	Inpatients n = 1123 with a mean age of 81 ± 7.5 years	Cognitive status (MMSE)<br>Functional status (ADL)	4	Poor	0	1	0	0
1	ACB	Cohort study	2018	Campbell et al.	Anticholinergics Influence Transition from Normal Cognition to Mild Cognitive Impairment in Older Adults in Primary Care	Community-dwelling people,<br>n = 350 with a mean age of 71.2 ± 5.1 years	Transition from normal to MCI in patients w/o dementia	2a	Good	0	1	0	0
1	ARS	Cohort study	2018	Clarke et al.	Association Between Objectively Measured Physical Activity and Opioid, Hypnotic, or Anticholinergic Medication Use in Older People: Data from the Physical Activity Cohort Scotland Study	Older people from the Physical Activity Cohort Scotland - Community-dwelling,<br>n = 310 with a mean age 77.3 ± 7 years	Physical activity	4	Poor	0	0	0	0
1	ACB	Cohort study	2018	Gamble et al.	Baseline anticholinergic burden from medications predicts incident fatal and non-fatal stroke in the EPIC-Norfolk general population	Outpatients,<br>n = 21'722 with a mean age of 58.9 ± 9.2 years	Incident stroke<br>Stroke mortality	2b	Good	0	0	1	0
3	ACB, ADS, ARS	Cohort study (in an intervention study)	2018	Jaïdi et al.	Threshold for a Reduction in Anticholinergic Burden to Decrease Behavioral and Psychological Symptoms of Dementia	Inpatients with dementia and hospitalized for behavioral and psychological symptoms (BPSD),<br>n = 147 with mean age of 84.1 ± 5.2 years	Clinical and statistical significant improvement in BPSD when ACH load is reduced	2a	Good	0	0	0	0
1	ARS	Cohort study	2018	Kose et al.	Assessment of aspiration pneumonia using the Anticholinergic Risk Scale	Patients from a rehabilitation ward, n = 618 with a median age of 79 years<br>with ACH: n = 162 with a median age of 80 years and without ACH: n = 456 with a median age of 78 years	Aspiration pneumonia	2b	Good	0	0	0	0
1	ARS	Cohort study	2018	Kose et al.	Anticholinergic load negatively correlates with recovery of cognitive activities of daily living for geriatric patients after stroke in the convalescent stage	Geriatric patients from a rehabilitation center,<br>n = 418 patients with median age of 78 years	Functional recovery after stroke	2b	Good	0	0	0	0
1	ACB	Cohort study (multicenter)	2018	Lattanzio et al.	Anticholinergic burden and 1-year mortality among older patients discharged from acute care hospital	Patients discharged from acute care hospitals,<br>n = 807 with a mean age of 81.0 ± 7.4 years	Mortality	2a	Good	0	0	1	0
1	ADS	Cohort study (for part of outcomes)	2018	Sevilla-Sanchez et al.	Prevalence, risk factors and adverse outcomes of anticholinergic burden in patients with advanced chronic conditions at hospital admission	Inpatients requiring palliative care,<br>n = 235 with a mean age of 86.8 ± 5.37 years	Days of hospital stay<br>Destination after discharge<br>Inhospital mortality<br>1-year survival	2a	Good	0	0	1	0
1	ACB	Cohort study	2018	Tan et al.	Anticholinergic burden and risk of stroke and death in people with different types of dementia	Patients with different dementia subtypes,<br>n = 39'107 with a mean age of 79.9 ± 7.9 years	Stroke<br>All-cause mortality	2b	Good	0	0	1	0
1	ADS	Cohort study	2018	Tiisanoja et al.	Anticholinergic burden and dry mouth among Finnish, community-dwelling older adults	Community-dwelling, non-smoking, dentate patients,<br>n = 152 with a mean age of 79.4 ± 3.67 years	Xerostomia<br>Low unstimulated salivary secretion	4	Poor	0	0	0	0
1	ACB	Cohort study	2018	Ziad et al.	Anticholinergic drug use and cognitive performances in middle age: findings from the CONSTANCES cohort	Participants living in France,<br>n = 34'267 aged 45-70 years	Cognitive performance (episodic memory, verbal fluency, executive functions)	2b	Good	0	1	0	0
1	ACB	Cohort study	2019	Ah et al.	Effect of anticholinergic burden on treatment modification, delirium and mortality in newly diagnosed dementia patients starting a cholinesterase inhibitor:<br>A population-based study	Patients with cholinesterase inhibitor treatment for dementia, n = 7438 aged > 60 years<br>ACB >3: n = 1554<br>ACB $\leq$ 1: n = 5884	Delirium (ICD-10 F5.0)<br>Mortality	2b	Good	1	0	1	0
4	ACB, ADS, ARS, DS	Cohort study (reanalysis on a RCT)	2019	Andre et al.	Anticholinergic exposure and cognitive decline in older adults: effect of anticholinergic exposure definitions in a 3-year analysis of the multidomain Alzheimer preventive trial (MAPT) study	Community-dwelling French adults,<br>n = 1396 with a mean age of 75.2 ± 4.4 years	Cognitive decline (assessed with a composite score following MMSE, Free and Cued Selective Reminding Test, Category Naming Test, Digit Symbol Substitution Test)	2a	Good	0	1	0	0
1	ACB	Cohort study (multicenter)	2019	Corsonello et al.	The excess mortality risk associated with anticholinergic burden among older patients discharged from acute care hospital with depressive symptoms	Hospitalized patients in 7 acute care centers,<br>n = 576 with a mean age of 79.6 ± 7.0 years	Mortality at 1-year after discharge	2a	Good	0	0	1	0
1	ACB	Cohort study	2019	Green et al.	Drugs Contributing to Anticholinergic Burden and Risk of Fall or Fall-Related Injury among Older Adults with Mild Cognitive Impairment, Dementia and Multiple Chronic Conditions: A Retrospective Cohort Study	Patients with impaired cognition,<br>n = 10'698 with a mean age of 79.1 ± 7.99 years	Falls<br>Falls related injuries	2b	Good	0	0	0	1
1	ACB	Cohort study (in a RCT)	2019	Joshi et al.	Verbal learning deficits associated with increased anticholinergic burden are attenuated with targeted cognitive training in treatment refractory schizophrenia patients	Schizophrenic patients, intervention group n = 24 with a mean age of 34.54 ± 12.13 years and control group n = 22 with a mean age of 35.73 ± 13.0 years	Learning verbal training in intervention group<br>Learning verbal training in control group	4	Poor	0	0	0	0
1	ACB	Cohort study	2019	Szabo et al.	Association between cumulative anticholinergic burden and falls and fractures in patients with overactive bladder: US-based retrospective cohort study	Outpatients with overactive bladder,<br>n = 154'432 with a mean age of 55.7 years	Falls<br>Fractures	2b	Good	0	0	0	1

Quality of anticholinergic burden scales and their impact on clinical outcomes - a systematic review, EJCP, Lisibach A et al, Corresponding author: Pr Chantal Csajka ,Center for Research and Innovation in Clinical Pharmaceutical Sciences, Rue du Bugnon 17, 1005 Lausanne

Appendix 6b: Identified validation studies (total n=104).

Number of compared ABS	ABS	Study design	Year	Author	Title	Study population	Clinical outcome	Evidence level (adapted EBM Oxford)	Quality (NOS, RoB 2.0)	Delirium	Cognition	Mortality	Falls
1	ADS	Nested Case-Control study	2016	Chatterjee et al.	Anticholinergic Medication Use and Risk of Dementia Among Elderly Nursing Home Residents with Depression	Uses 2007 to 2010 Minimum Data Set linked Medicare data set from all states (US), n =191'304 with depression and no baseline dementia, incidence matched cohort n = 141'940 with n = 28'388 cases and n = 113'552 controls, mean age of 80 years	Dementia	3	Good	0	0	0	0
2	ACB, ADS	Nested Case-Control study	2016	Chatterjee et al.	Anticholinergic Medication Use and Risk of Fracture in Elderly Adults with Depression	Uses 2007 to 2010 Minimum Data Set linked Medicare data set from all states (US), n = 352'937 with depression and no baseline fractures or falls in 2007, incidence matched cohort n = 202'260 with n = 161'808 controls and n = 40'452 cases, mean age of 81.4 ± 7.4 years	Fracture risk	3	Good	0	0	0	0
1	ACB	Case-Control study	2016	Zia et al.	Anticholinergic burden is associated with recurrent and injurious falls in older individuals	Patients, n = 263 cases with a mean age of 75.3 ± 7.3 years and n = 165 controls with a mean age of 72.13 ± 5.5 years	Recurrent and injurious falls	3	Good	0	0	0	1
1	ADS	Nested Case-Control study	2017	Chatterjee et al.	Risk of Mortality Associated with Anticholinergic Use in Elderly Nursing Home Residents with Depression	Uses 2007 to 2010 Minimum Data Set linked Medicare data set from all states (US), n = 433'812 with depression, incidence matched cohort n = 224'740 with n= 179'792 controls and n = 44'948 cases, mean age of 83 years	Risk of mortality	3	Good	0	0	1	0
1	ADS	Nested Case-Control study	2017	Lampela et al.	Anticholinergic Exposure and Risk of Pneumonia in Persons with Alzheimer's Disease: A Nested Case-Control Study	Community-dwelling patients diagnosed with AD, cases n = 12'442 with a mean age of 83.3 ± 6.7 years, controls n = 24'349 with a mean age of 83.3 ± 6.5 years	Risk of pneumonia	3	Good	0	0	0	0
1	ADS	Case-Control study (multicenter)	2018	Aldebert et al.	Association of Anticholinergic Drug Use With Risk for Late Age-Related Macular Degeneration	Patients from 4 French ophthalmologic centers, n = 400 with cases n = 200 with a mean age of 74.8 ± 9.2 years and controls n = 200 with a mean age of 75.5 ± 7.2 years	Late Age-Related Macular Degeneration	4	Poor	0	0	0	0
1	ARS	Nested Case-Control study	2018	Kose et al.	Anticholinergic drugs use and risk of hip fracture in geriatric patients	Patients from a rehabilitation ward, n = 601 with a median age of 79 years, fracture group n = 68 with a median age of 80.5 years and non-fracture group n = 533 with a median age of 79 years	Hip fracture	3	Fair	0	0	0	0
1	ARS	Case-Control study	2018	Machado-Duque et al.	Drugs With Anticholinergic Potential and Risk of Falls With Hip Fracture in the Elderly Patients: A Case-Control Study	Outpatients, cases n = 300 and controls n = 600 with a mean age of 81.6 years	Falls with hip fracture	3	Good	0	0	0	1
1	ACB	Nested Case-Control study	2018	Richardson et al.	Anticholinergic drugs and risk of dementia: case-control study	Outpatients, aged ≥ 65 years n = 40'770 cases and n = 283'933 controls (matching 1:7)	Incidence of dementia	4	Poor	0	0	0	0
1	CI, PI	Cross-sectional	2004	Minzenberg et al.	Association of anticholinergic load with impairment of complex attention and memory in schizophrenia	Outpatients with schizophrenia or schizoaffective disorders n = 106 with a mean age of 39.9 ± 11.3 years	Simple attention<br>Complex attention<br>Short-term memory	5	Poor	0	1	0	0
1	ACB	Cross-sectional	2009	Kolanowski et al.	A Preliminary Study of Anticholinergic Burden and Relationship to a Quality of Life Indicator, Engagement in Activities, in Nursing Home Residents With Dementia	Nursing home residents with dementia, n = 87 with a mean age of 85.7 ± 6.3 years	Quality of life: Multiple engagement observations	5	Good	0	0	0	0
1	ARS	Cross-sectional	2011	Lowry et al.	Clinical and demographic factors associated with antimuscarinic medication use in older hospitalized patients	Inpatients, n = 362 with a mean age of 83.6 ± 6.6 years	Institutionalization and comorbidities	5	Poor	0	0	0	0
1	ARS	Cross-sectional	2011	Teramura-Gronblad et al.	Use of Anticholinergic Drugs and Cholinesterase Inhibitors and Their Association with Psychological Well-Being Among Frail Older Adults in Residential Care Facilities	Nursing homes, n = 1475 with a mean age of 81.7 ± 7.6 years	Psychological well-being	5	Poor	0	0	0	0
1	ADS	Cross-sectional	2012	Drag et al.	Prescribing Practices of Anticholinergic Medications and Their Association With Cognition in an Extended Care Setting	Inpatients non-demented and non-delirious, n = 450 with a mean age of 67.9 ± 10.5 years	Cognitive function	5	Poor	0	1	0	0
1	ARS	Cross-sectional	2013	Bostock et al.	Associations between different measures of anticholinergic drug exposure and Barthel Index in older hospitalized patients	Consecutive series of community-dwelling and institutionalized settings, acute geriatric admission, n = 271 with a mean age of 83 ± 7 years	Barthel Index (physical function)<br>AMT (Abbreviated Mental Test)	5	Good	0	1	0	0
1	ADS	Cross-sectional	2013	Kersten et al.	Higher anticholinergic drug scale (ADS) scores are associated with peripheral but not cognitive markers of cholinergic blockade. Cross sectional data from 21 Norwegian nursing homes	Nursing home residents, n = 87 with a mean age of 73 years	Cognitive function (MMSE)<br>Functional outcome (ADL)	5	Poor	0	1	0	0
3	ADS, ARS, Chew	Cross-sectional	2013	Lampela et al.	Anticholinergic Drug Use, Serum Anticholinergic Activity, and Adverse Drug Events Among Older People: A Population-Based Study	Community-dwelling, n = 621 with a mean age of 81.7 ± 4.9 years	Adverse events<br>Cognitive function (MMSE, GDP)<br>Functional outcome (ADL, IADL)	5	Poor	0	1	0	0

Quality of anticholinergic burden scales and their impact on clinical outcomes - a systematic review, EJCP, Lisibach A et al, Corresponding author: Pr Chantal Csajka ,Center for Research and Innovation in Clinical Pharmaceutical Sciences, Rue du Bugnon 17, 1005 Lausanne													
**Appendix 6b:** Identified validation studies (total n=104).													
**Number of compared ABS**	**ABS**	**Study design**	**Year**	**Author**	**Title**	**Study population**	**Clinical outcome**	**Evidence level** (adapted EBM Oxford)	**Quality** (NOS, RoB 2.0)	**Delirium**	**Cognition**	**Mortality**	**Falls**
2	ACB, ARS	Cross-sectional	2013	Pasina et al.	Association of anticholinergic burden with cognitive and functional status in a cohort of hospitalized elderly: comparison of the anticholinergic cognitive burden scale and anticholinergic risk scale: results from the REPOSI study	Hospitalized patients, n = 1232 with age of ≥ 65 years	Cognitive function (SBT)<br>Physical function (BI)	5	Poor	0	1	0	0
1	ACB	Cross-sectional	2014	Lanctot et al.	Assessing Cognitive Effects of Anticholinergic Medications in Patients With Coronary Artery Disease	Outpatients with coronary artery disease, n = 131 with a mean age of 64.2 ± 9.1 years	Attention, speed, executive function	5	Poor	0	0	0	0
1	ADS	Cross-sectional	2015	Moulis et al.	Exposure to Atropinic Drugs and Frailty Status	Patients attending a geriatric frailty clinic, n = 437 with a mean age of 83.05 ± 6.15 years	Frailty	5	Poor	0	0	0	0
1	ACB	Cross-sectional	2016	O'Dwyer et al.	Association of anticholinergic burden with adverse effects in older people with intellectual disabilities: an observational cross-sectional study	Patients with intellectual disability, n = 736 aged ≥ 40 years	Daytime dozing<br>Constipation	5	Poor	0	0	0	0
2	ACB, ADS	Cross-sectional	2017	Ang et al.	The Impact of Medication Anticholinergic Burden on Cognitive Performance in People With Schizophrenia	Outpatients and inpatients with schizophrenia, n = 705 with a mean age of 39.18 ± 9.71 years	Cognitive performance (executive functions, fluency/memory, speed/vigilance)	5	Poor	0	1	0	0
5	ACB, ADS, ARS, Chew, CrAS	Cross-sectional	2017	Dauphinot et al.	Anticholinergic drugs and functional, cognitive impairment and behavioral disturbances in patients from a memory clinic with subjective cognitive decline or neurocognitive disorders	Older outpatients visiting memory clinic, n = 473 with a mean age of 80.58 ± 7.48 years	Functional impairment (Functional and global cognitive performances, as well as the behavioral and psychological symptoms of dementia)	5	Poor	0	1	0	0
1	ADS	Cross-sectional	2017	Eum et al.	Cognitive burden of anticholinergic medications in psychotic disorders	Patients with schizophrenia, schizoaffective and bipolar disorders, n = 483 with a mean age 36 years	Cognitive function (BACS) if ADS ≥4	5	Poor	0	1	0	0
8	ACB, ADS, ARS, ABC, ACL, Chew, CrAS, CABS	Cross-sectional	2017	Mayer et al.	Comparison of Nine Instruments to Calculate Anticholinergic Load in a Large Cohort of Older Outpatients: Association with Cognitive and Functional Decline, Falls, and Use of Laxatives	Home-dwelling patients, n = 2761 with a mean age of 72 ± 6 years	Cognitive impairment (MMSE)<br>Functional decline (Barthel Index)<br>Falls<br>Use of laxatives	5	Poor	0	1	0	1
1	ACB	Cross-sectional	2017	Pfistmeister et al.	Anticholinergic burden and cognitive function in a large German cohort of hospitalized geriatric patients	Hospitalized patients, n = 89'579 with a median age of 82 years, complete case analysis for cognitive impairment n = 59'007 and for dementia n = 68'388	Cognitive Impairment (MMSE)<br>Dementia	5	Poor	0	1	0	0
1	ACB	Cross-sectional	2017	Tsoutsoulas et al.	Anticholinergic Burden and Cognition in Older Patients With Schizophrenia	Community-dwelling patients with schizophrenia or schizoaffective disorder, n = 60 with a mean age of 63.6 ± 6.83 years	Alzheimer's dementia-related cognitive functions	5	Poor	0	1	0	0
1	ACB	Cross-sectional	2018	Ablett et al.	A high anticholinergic burden is associated with a history of falls in the previous year in middle-aged women: findings from the Aberdeen Prospective Osteoporosis Screening Study	Community-dwelling women, n = 3883 with a mean age of 54.33 ± 2.27 years	History of falls	5	Poor	0	0	0	1
1	ACB	Cross-sectional	2019	Pasina et al.	Relation Between Delirium and Anticholinergic Drug Burden in a Cohort of Hospitalized Older Patients: An Observational Study	Inpatients, n = 502, with delirium n = 151 with a mean age of 85.0 ± 6.5 years, without delirium n = 32 with a mean age of 83.4 ± 6.5 years	Delirium (4 AT)	5	Poor	1	0	0	0
